# RETRACTION: Knowledge, Attitude, Practice, and Associated Factors of Implant Use in Women, Ethiopia

**DOI:** 10.1155/bmri/9804791

**Published:** 2025-10-26

**Authors:** BioMed Research International

RETRACTION: K. T. Tegegne, G. Teferi, T. K. Wudu, Y. Abinew, E. T. Tegegne and M. K. Tessema, “Knowledge, Attitude, Practice, and Associated Factors of Implant Use in Women, Ethiopia,” *BioMed Research International*, 2024 (2024): 9978336, https://doi.org/10.1155/2024/9978336.

The above article, published online on 22 February 2024 in Wiley Online Library (wileyonlinelibrary.com), has been retracted by agreement between the handling editorial board member, Carlos M. Ardila, and John Wiley & Sons Ltd. The retraction has been agreed due to high similarity to a previously published article [1], which was not appropriately cited. Separately, the authors also requested to retract the article due to disputed authorship and Girma Teferi informed the journal that they have no knowledge of the submission to *BioMed Research International*.
